# Molecular xenomonitoring reveals *Anopheles funestus* and *An. rivulorum* as the primary vectors of lymphatic filariasis in coastal Kenya

**DOI:** 10.1186/s13071-024-06513-0

**Published:** 2024-10-09

**Authors:** Brian Bartilol, Lawrence Babu, Karisa Garama, Jonathan Karisa, Alice Kamau, Charles Mwandawiro, Caroline Wanjiku, Charles Mbogo, Marta Maia, Joseph Mwangangi, Martin Kibet Rono

**Affiliations:** 1 KEMRI-Centre for Geographic Medicine Research Coast, Kilifi, Kenya; 2 KEMRI-Wellcome Trust Research Programme, Kilifi, Kenya; 3 Liverpool School of Tropical Medicine, Liverpool, UK; 4 Eastern and Southern Africa Centre of International Parasite Control, Nairobi, Kenya; 5 Pan-African Mosquito Control Association, Nairobi, Kenya; 6 Centre for Global Health and Tropical Medicine, University of Oxford, Oxford, UK; 7 Pwani University Bioscience Research Centre, Kilifi, Kenya

**Keywords:** Lymphatic filariasis, *Anopheles funestus*, Kenya, Xenomonitoring

## Abstract

**Background:**

Lymphatic filariasis (LF) is an infectious neglected tropical disease caused by mosquito-borne nematodes such as *Wuchereria bancrofti*, *Brugia malayi*, and *Brugia timori*. Globally, LF affects 51 million people, with approximately 863 million at risk in 47 countries. In Kenya, filariasis is endemic along the entire coastal strip, and more recently, at the Kenya–Ugandan border. The World Health Organization (WHO) recommends mass drug administration to reduce disease transmission and morbidity. Monitoring the effectiveness of such interventions relies on robust surveillance, achieved through microscopic examination of microfilariae in nighttime blood, detection of circulating filarial antigens (CFA), and molecular xenomonitoring. We focused on molecular xenomonitoring along the Kenyan coast due to its noninvasive nature and the opportunity to identify new vectors.

**Methods:**

In 2022, mosquitoes were collected from Kilifi, Kwale, and Taita-Taveta counties located within the LF endemic region in Kenya. Subsequently, genomic deoxyribonucleic acid (gDNA) was extracted from these mosquitoes for speciation and analysis of *Wuchereria bancrofti* infection rates. The impact of sociodemographic and household attributes on infection rates was assessed using generalized estimating equations.

**Results:**

A total of 18,121 mosquitoes belonging to *Culicinae* (63.0%, *n* = 11,414) and *Anophelinae* (37.0%, *n* = 6707) subfamilies were collected*.* Morphological identification revealed that Anopheline mosquitoes were dominated by *An. funestus* (45.4%, *n* = 3045) and *An. gambiae* (42.8%, *n* = 2873). *Wuchereria bancrofti* infection rates were highest in Kilifi (35.4%; 95% CI 28.0–43.3%, *n* = 57/161) and lowest in Taita Taveta (5.3%; 95% CI 3.3–8.0%, *n* = 22/412). The major vectors incriminated are *An. rivulorum, An. funestus* sensu stricto, and *An. arabiensis*. Mosquitoes of the *An. funestus* complex were significantly associated with LF transmission (OR 18.0; 95% CI 1.80–180; *p* = 0.014). Additionally, a higher risk of transmission was observed outdoors (OR 1.74; 95% CI 1.08–2.82; *p* = 0.024) and in homesteads that owned livestock (OR 2.00; 95% CI 1.09–3.66; *p* = 0.025).

**Conclusions:**

In this study, we identified *An. funestus* s.l. sibling species, *An. rivulorum* and *An. funestus* s.s., as the primary vectors of lymphatic filariasis along the Kenyan coast. These findings also highlight that a significant portion of disease transmission potentially occurs outdoors where indoor-based vector control tools, including long-lasting insecticidal nets and indoor residual spray, may not be effective. Therefore, control measures targeting outdoor resting mosquitoes such as zooprophylaxis, larval source management, and attractive sugar baits may have potential for LF transmission reduction.

**Graphical Abstract:**

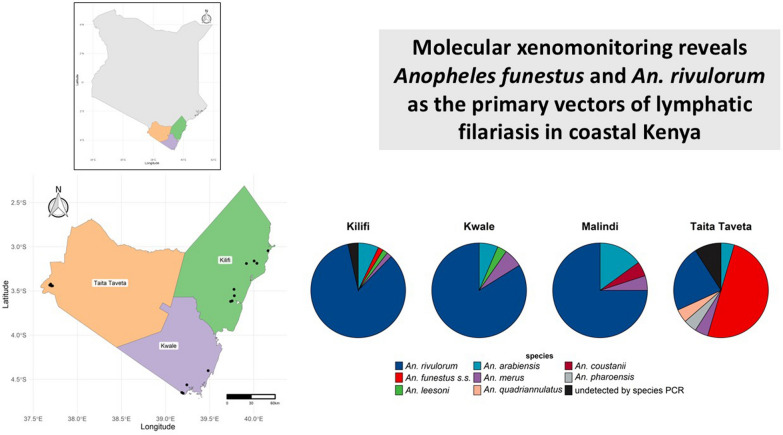

## Background

Lymphatic filariasis (LF) is an infectious neglected tropical disease caused by mosquito-borne nematodes such as *Wuchereria bancrofti, Brugia malayi*, and *Brugia timori*. Globally LF accounts for 51 million cases with approximately 863 million people in 47 countries still at risk of infection [1]. Clinical symptoms of LF include hydrocele, lymphedema, and adenolymphangitis. At an advanced stage, lymphedema develops into elephantiasis, which is characterized by swollen body parts (mainly legs, genitals, arms, and breasts) and disfiguration that results in sociopsychological problems for patients and their families. In sub-Saharan Africa, filariasis is transmitted to humans by mosquitoes of the genera *Anopheles* and *Culex*. In urban areas, transmission is mainly carried out by *Culex quinquefasciatus*, whereas in rural areas it is dominated by *An. funestus* s.l. and *An. gambiae* s.l. mosquitoes [2]. Transmission occurs through bites from female mosquitoes infected with L3 larvae, which develop from microfilariae ingested from infected humans. Once they penetrate the skin, the L3 larvae migrate to the lymphatic system where they mature into adult worms, causing disruption in normal circulation leading to clinical symptoms previously described. The worms also produce microfilariae that migrate back to the blood stream and get ingested by a mosquito during a subsequent blood meal perpetuating the transmission cycle. In Kenya, filariasis is endemic along the coastal region [3–9] and has recently been reported further inland in Busia County, located at the Kenyan–Ugandan border [10]. The main vectors of LF in Kenya are *An. gambiae* s.l., *An. funestus* s.l., and *Cx. quinquefasciatus*, with varying transmission intensities attributed to diverse ecological and environmental conditions [6, 8, 11–13].

In 2002, the World Health Organization (WHO) launched the Global Programme to Eliminate Lymphatic Filariasis (GPELF) with the ambitious target of eliminating LF by 2020 through mass drug administration (MDA) [1]. Co-administration of albendazole (400 mg) and diethylcarbamazine citrate (DEC) (6 mg/kg) was recommended by the WHO for all eligible individuals in filariasis-endemic areas to reduce transmission and disease morbidity. New treatment guidelines recommend a triple therapy regimen consisting of diethylcarbamazine, albendazole, and ivermectin in countries without onchocerciasis. Mass drug administration has been tremendously successful, leading to a 74% decline in LF globally. Kenya initiated LF elimination efforts in 2002 through annual MDA campaigns using DEC and albendazole. MDA began in Kilifi district; a known LF foci followed by scale-up campaigns in Kwale and Malindi districts in 2003 and subsequently to Tana River, Taita-Taveta, and finally in Mombasa [7].

The success of this strategy relies on robust surveillance and monitoring of parasite infection. Tracking data on local populations of filariasis-transmitting vectors provides an opportunity for monitoring disease transmission dynamics. Monitoring MDA performance is mainly achieved through microscopic examination of microfilariae in nighttime blood and detection of circulating filarial antigens (CFA). Although microscopic examinations provide the most reliable estimates, nighttime sampling is a major challenge, and infections may be missed in presence of unmated adult worms [14–16]. While monitoring CFA can provide accurate information about the prevalence of *W. bancrofti* infection, antibody testing offers a sensitive indicator of exposure levels but cannot distinguish between previous and current infections, potentially leading to an overestimation of the true burden of infection. Molecular xenomonitoring (MX) that relies on polymerase chain reaction (PCR) has been suggested by the WHO as an important noninvasive surveillance tool to complement human surveys [16, 17]. MX provides a platform for monitoring infection in known vectors and provides an opportunity to incriminate new vectors involved in the transmission of *W. bancrofti* [18]. The present paper reports LF surveillance in adult mosquitoes collected on the Kenyan coast.

## Methods

### Study area

**Figure 1:**
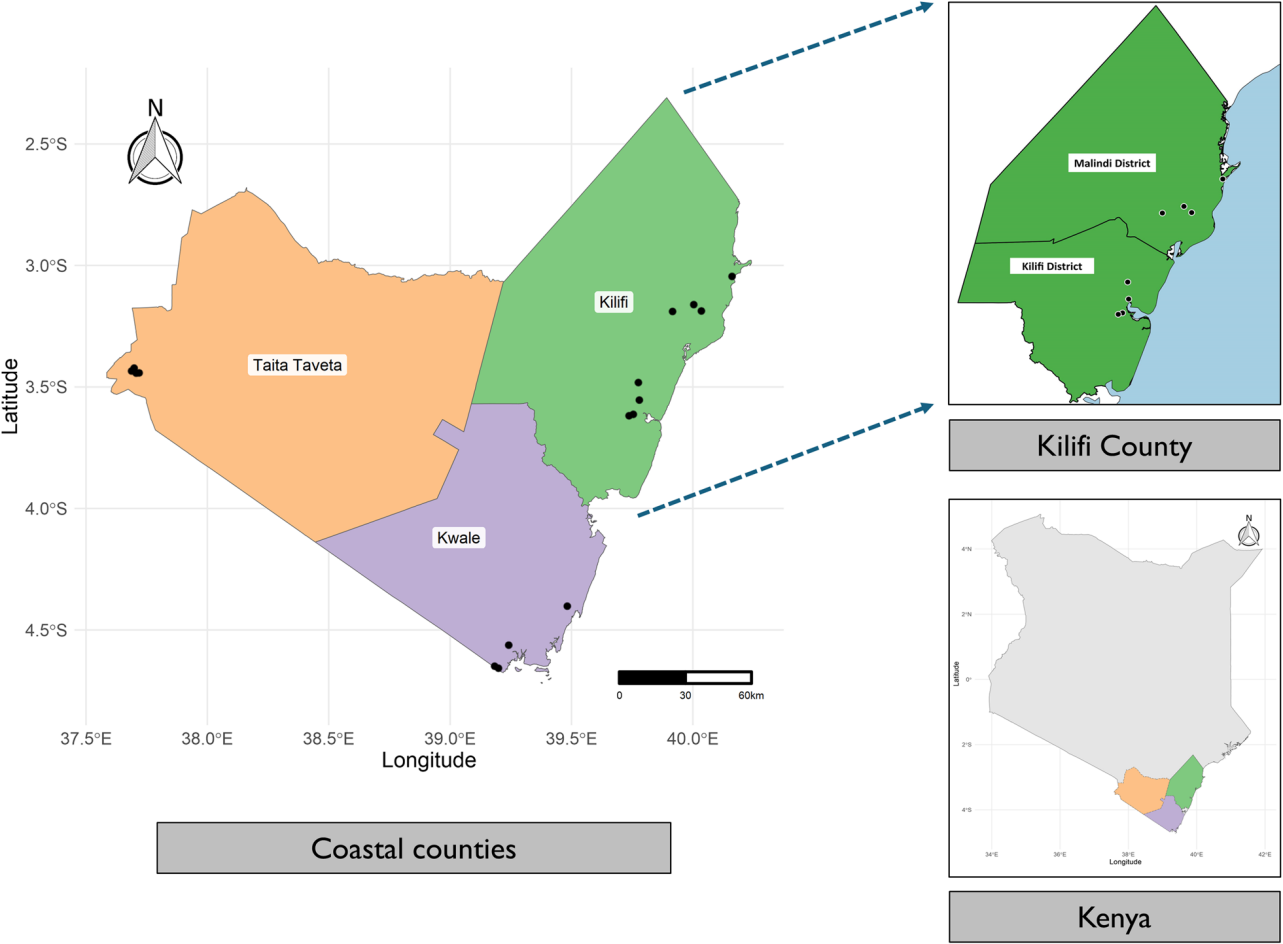
A map showing mosquito sampling sites. Sampling in Kilifi County was divided into two districts: Kilifi and Malindi

The study was conducted in the selected sites of Kilifi, Kwale, and Taita-Taveta counties along the Kenyan coast (Fig. 1). These three counties experience a moderately hot (21–31 °C) and moist (> 1000 mm precipitation per year) climate and have a combined population of approximately 2.4 million people. Climatic changes observed in recent years include delays in the onset of rains, reduction in water volume or drying up of wells and rivers, and increases in temperatures [19–21]. Despite shared climatic conditions, mosquito composition and abundance are heterogeneous, with a notable decline in the *An. gambiae* s.s. population [22]. A total of 16 sites were selected for vector sampling (Fig. 1). In Kilifi County, sampling was conducted in two former administrative units, Kilifi and Malindi district, because MDA activities had previously been carried out extensively in the two regions.

### Mosquito collection

This cross-sectional study involved mosquito collection both indoors and outdoors in 10 households at each of the 16 sites using Centers for Disease Control and prevention light traps (CDC-LT). Sampling was carried out during the dry season (January, February, and March) and at the end of the wet season (July) in 2022. The traps were set at dusk (1800 h) and collected at dawn (0600 h) on the next day. Geo-reference coordinates were collected using the eTrex^®^ 10 (Garmin, Kansas, USA). Indoor traps were set in houses where at least one member of the household person spent the night, while the outdoor traps were placed next to livestock sheds or within a distance no more than 5 m from the house containing the indoor trap. The collected mosquitoes were identified using the morphological keys of Gillies and Coetzee [23], sorted by sex and physiological state, and then counted. All anopheline mosquitoes were preserved individually in micro centrifuge tubes containing a desiccant (silica pellets) and transported to the Kenya Medical Research Institute-Wellcome Trust Research laboratory for further analysis. A small proportion of the culicine mosquitoes were archived, and the rest were discarded.

### Mosquito dissection

Using sterile scalpels and forceps, the adult female anopheline mosquitoes were dissected into two parts: head/thorax and abdomen, and stored at −80 °C.

### DNA extraction

Genomic deoxyribonucleic acid (gDNA) was extracted from the mosquito head and thorax as previously described, with minor modifications [24]. Briefly, sterile tungsten beads were transferred into the 1.5 ml microcentrifuge tubes and topped up with 100 µl of 10% chelex and lysed using a tissue lyser at 30 Hz for 1 min. The beads were removed from the tubes and the lysate incubated at 100 °C for 10 min in a Thermomixer (Eppendorf, Hamburg, Germany). The solution was then centrifuged at 10,000 × *g* for 2 min, and supernatant was transferred to a new microcentrifuge tube and stored at −80 °C.

### Molecular identification of *Anopheles* gambiae and *Anopheles* funestus sibling species

Diagnostic polymerase chain reaction (PCR) for *An. gambiae* sibling species was done using primers targeting the intergenic spacer (IGS) region of the ribosomal DNA [25]. For *An. funestus*, PCR primers targeting the internal transcribed spacer region 2 (ITS2) were used [26]. Each PCR reaction consisted of 4 µL 5X Green GoTaq^®^ Flexi Buffer, 2.4 µL magnesium chloride, 4 µL nuclease free water, 0.5 µL deoxynucleoside triphosphates (dNTPs), 0.1 µL GoTaq^®^ G2 Flexi DNA Polymerase, 1 µL of each primer, and 4 µL of the mosquito DNA template. Thermocycling conditions consisted of an enzyme activation step at 95 °C for 5 min, followed by 35 cycles of denaturation at 95 °C for 15 s, annealing at 55 °C for 20 s, extension at 72 °C for 30 s, and a final elongation step at 72 °C for 10 min. PCR amplicons were resolved on a 1.5% agarose gel stained using RedSafe^™^ Nucleic Acid Staining Solution (iNtRON Biotechnology, Korea) and visualized using the ChemiDoc Imaging System (Bio-Rad, USA) to resolve the different species.

### Detection of *Wuchereria bancrofti*

*Wuchereria bancrofti* was detected using the method described by Zhong et al. [27] with minor modifications. The PCR primers target the genus-specific, multicopy (~ 300 copies) *Ssp I* repeat DNA family. The PCR reaction consisted of 4 µL 5X Green GoTaq^®^ Flexi Buffer, 2.4 µL magnesium chloride, 7 µL nuclease free water, 0.5 µL deoxynucleoside triphosphates (dNTPs), 0.1 µL GoTaq^®^ G2 Flexi DNA Polymerase, 1 µL of each primer, and 4 µL of the mosquito DNA. The cycling conditions consisted of an initial enzyme activation step at 95 °C for 5 min, followed by 35 cycles of denaturation at 95 °C for 15 s, annealing at 55 °C for 20 s, extension at 72 °C for 30 s, and a final elongation step at 72 °C for 10 min. Amplicons were resolved on a 1.5% agarose gel stained with RedSafe™ Nucleic Acid Staining Solution (iNtRON Biotechnology, Korea) and visualized on the ChemiDoc Imaging System (Bio-Rad, USA). Samples with a band size of 188 base pairs were identified as positive.

### Statistical analysis

Data were entered and cleaned in a Microsoft excel file. Statistical analysis and data visualization were conducted using R software, version 4.2.1 [28]. Infection proportions in the mosquito vectors were determined by dividing the number of *W. bancrofti* positive mosquitoes by the total number of mosquitoes analyzed per county in Kwale and Taita-Taveta, and per district in Kilifi County (Kilifi and Malindi). To assess the impact of various sociodemographic and household attributes on LF positivity, we employed a multilevel logistic regression model using generalized estimating equations (GEE) assuming a binomial distribution. The GEE approach was chosen to account for the correlated nature of repeated observations within the regions. The model was fitted using the geeglm function, with LF positivity as the binary outcome variable. The risk factor variables included season, site of mosquito collection, mosquito species, presence or absence of eaves, livestock, poultry, bed nets, type of material used in roofs and walls, and number of occupants. These factors have previously been shown to influence the transmission of vector-borne diseases [29–32]. We specified a logistic link function and selected an independent correlation structure. This was done to model within-region correlations considering the binary outcome of LF (positive or negative) and the clustered nature of the data. The results are reported as odds ratios (OR) along with 95% confidence intervals (CI) to quantify the association between the risk factor and LF positivity.

## Results

### Vector composition and abundance

**Figure 2:**
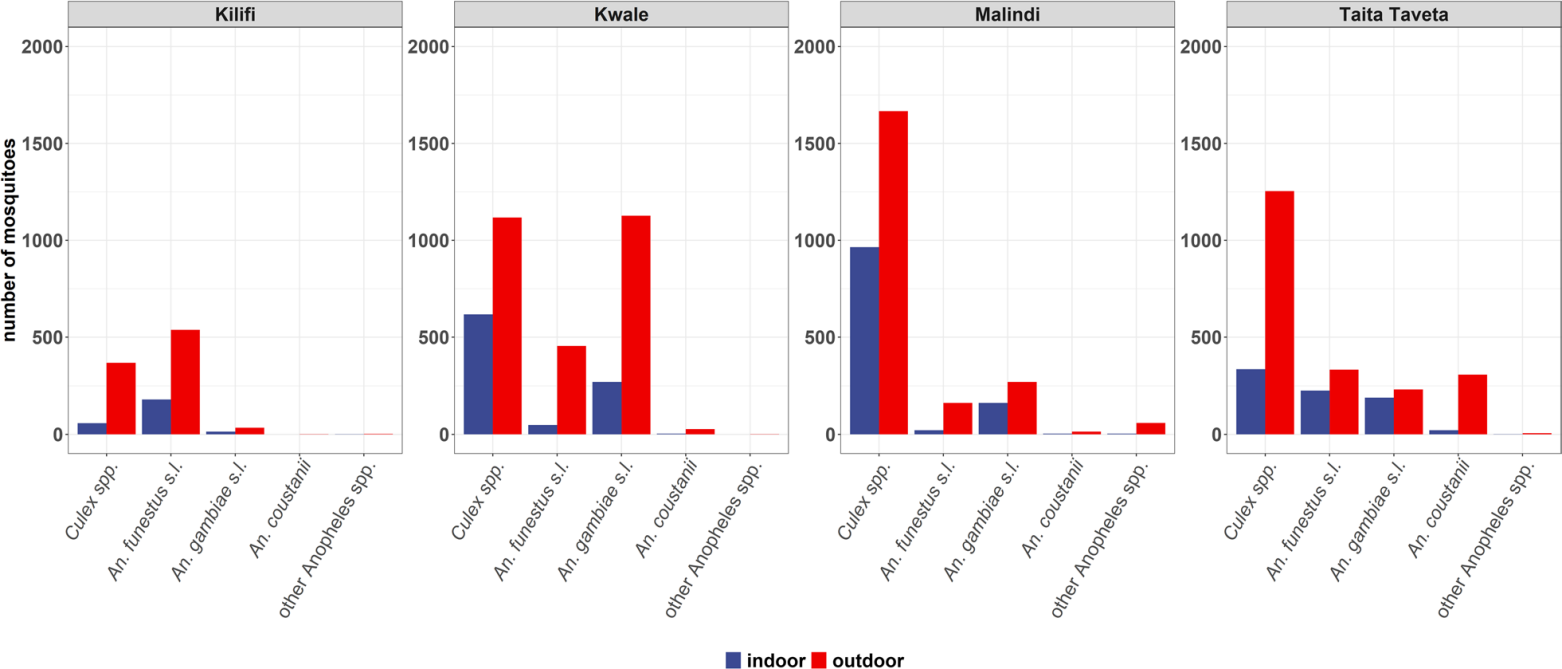
Proportion of mosquito collections indoors and outdoors

A total of 18,121 mosquitoes were collected from 16 sites (Table 1). They belonged to the *Culicinae* (*n* = 11,414, 63%) and *Anophelinae* (*n* = 6707, 37%) subfamilies, with most of them being caught outdoors (Fig. 2). Morphological identification revealed that *Anopheles* mosquitoes consisted of *An. funestus* s.l. (*n* = 3045, 45.4%), *An. gambiae* s.l. (*n* = 2873, 42.8%), *An. coustanii* (*n* = 662, 9.9%), *An. pharoensis* (*n* = 75, 1.1%), *An. maculpalpis* (*n* = 27, 0.4%), *An. pretoriensis* (*n* = 23, 0.3%), and *An. moucheti* (*n* = 2, 0.03%).

**Table 1:** Number of morphologically identified mosquitoes collected from the 16 sites along the Kenyan coast

County	Site	Season	*Culex spp*	*An. funestus*	*An. gambiae*	*An. coustanii*	*An. maculipalpis*	*An. moucheti*	*An. pharoensis*	*An. pretoriensis*
Kilifi	Garithe*	Dry	107	0	21	0	0	0	0	0
		Wet	334	0	184	0	0	0	0	0
	Jaribuni	Dry	279	142	24	0	0	0	0	0
		Wet	5	310	7	1	3	0	0	0
	Jilore*	Dry	343	168	63	6	0	0	5	0
		Wet	55	133	53	14	0	0	59	4
	Marana*	Dry	1828	4	109	0	0	0	0	0
		Wet	742	28	115	4	0	0	0	0
	Mbogolo*	Dry	352	0	86	0	0	0	0	0
		Wet	319	1	34	0	0	0	0	0
	Rare	Dry	12	3	0	0	0	0	0	0
		Wet	13	14	3	0	0	0	0	0
	Shibe	Dry	54	8	3	0	0	0	0	0
		Wet	44	3	2	0	0	0	0	0
	Sihu	Dry	80	466	21	0	0	0	1	0
		Wet	9	302	5	0	0	0	0	0
Kwale	Fihoni	Dry	91	0	1	0	0	0	0	1
		Wet	244	237	21	23	23	0	0	0
	Jego	Dry	374	442	1332	1	0	0	0	1
		Wet	484	24	86	8	0	0	0	0
	Kogeswa	Dry	329	22	24	0	0	0	0	0
		Wet	128	10	29	0	0	0	0	12
	Migombani	Dry	365	8	41	0	0	0	0	0
		Wet	879	12	53	0	0	0	0	0
Taita-Taveta	Kimundia	Dry	606	13	173	135	0	0	0	0
		Wet	556	83	57	150	0	0	3	0
	Kiwalwa	Dry	352	62	76	142	0	0	0	0
		Wet	64	277	26	30	0	0	2	0
	Mwarusa	Dry	1279	19	142	45	0	0	2	1
		Wet	555	77	30	72	1	0	1	0
	Njoro	Dry	336	54	31	8	0	0	0	4
		Wet	196	123	21	23	0	2	2	0

^*^These sites are located in what was formerly Malindi district

### Bancroftian filariasis infection rates

Infection rates varied across sites, with the highest observed in Kilifi (35.4%; 95% CI 28–43.3%, *n* = 57/161), followed by Kwale (11.7%; 95% CI 8.1–16.3%, *n* = 31/264), Malindi (8.3%; 95% CI 5.2–12.6%, *n* = 20/240), and Taita-Taveta (5.3%; 95% CI 3.4–8.0%, *n* = 22/412) (Table 2). In Kilifi, the highest proportions of *W. bancrofti*-infected mosquitoes were *An. funestus* s.l. (42.1%, *n* = 51) followed by *An. gambiae* s.l. (15.4%, *n* = 6). A similar trend was observed for Malindi, Kwale, and Taita Taveta.

**Table 2:** *Wuchereria bancrofti* prevalence rates in the three coastal counties. However, it is important to note that Kilifi County is divided into Kilifi and Malindi districts

District	Species	LF positive	Total tested	Infection rates
Kilifi	*An. funestus*	51	121	42.1
	*An. gambiae*	6	39	15.4
	*An. nili*	0	1	0.0
	Total	57	161	35.4
Kwale	*An. funestus*	27	123	22.0
	*An. gambiae*	4	138	2.9
	*An. maculpalpis*	0	3	0.0
	Total	31	264	11.7
Malindi	*An. coustanii*	1	5	20.0
	*An. funestus*	15	62	24.2
	*An. gambiae*	4	165	2.4
	*An. pharoensis*	0	8	0.0
	Total	20	240	8.3
Taita Taveta	*An. coustanii*	0	63	0.0
	*An. funestus*	18	121	14.9
	*An. gambiae*	3	222	1.4
	*An. pharoensis*	1	5	20.0
	*An. pretoriensis*	0	1	0.0
	Total	22	412	5.3

### Infection rates at sibling species level

**Figure 3:**
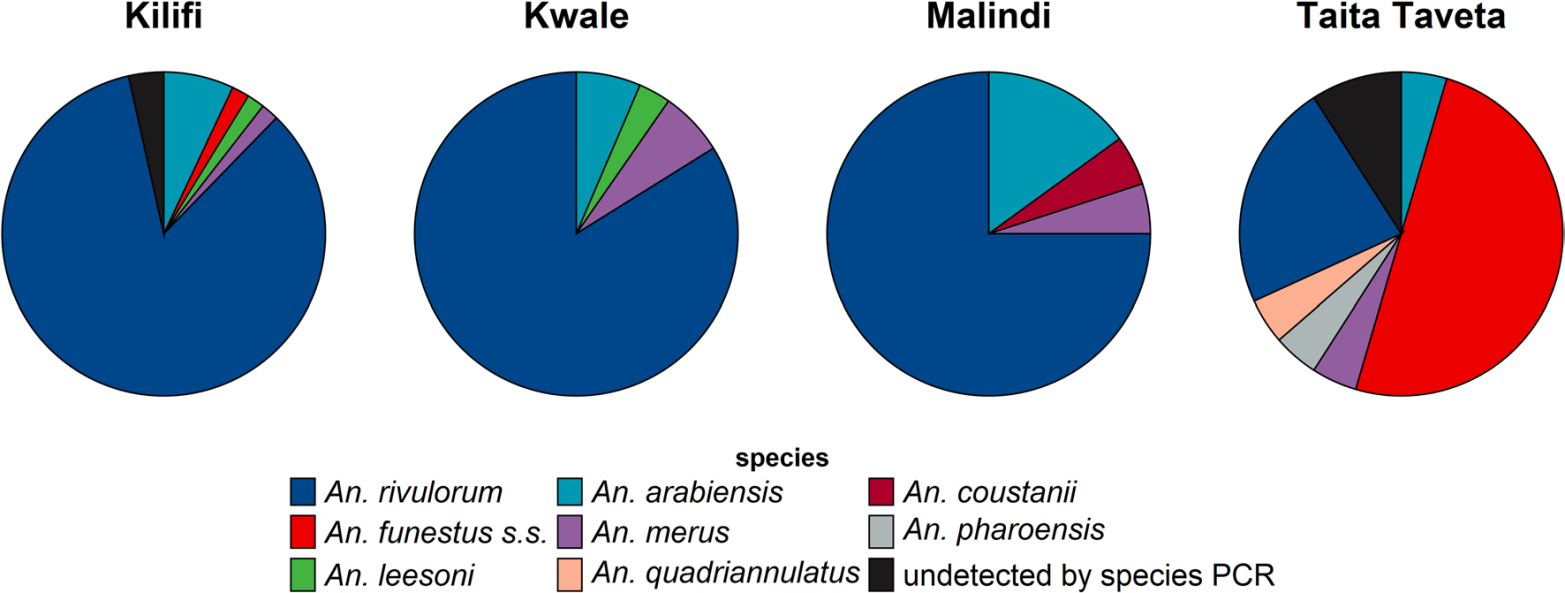
Distribution of Anopheles vectors infected with *W. bancrofti* in the Kenya coastal region

At the vector sibling species level, parasite infection rates were highest in *An. rivulorum* (8.7%, *n* = 94), followed by *An. funestus* s.s. (1.1%, *n* = 12), *An. arabiensis* (0.9%, *n* = 10), and *An. merus* (0.5%, *n* = 5) (Table 3). Notably, *An. rivulorum* is the dominant vector of LF in Kilifi, Kwale, and Malindi, whereas in Taita-Taveta it is *An. funestus* s.s. (*n* = 11) (Fig. 3).

**Table 3:** *Wuchereria bancrofti* infection rates in the mosquito vectors captured in the Kenyan coastal region

Species	*W. bacrofti* infection rates by PCR			
	Positive (*n*)	Negative (*n*)	Species infection rate (%)	Overall infection rate (%)
*An. rivulorum*^*2*^	94	193	32.8	8.7
*An. funestus* s.s.^*2*^	12	67	15.2	1.1
*An. arabiensis*^*2*^	10	359	2.7	0.9
*An. merus*^*2*^	5	91	5.2	0.5
*An. funestus not detected by PCR*^*2*^	3	18	14.3	0.3
*An. leesoni*^*2*^	2	23	8.0	0.2
*An. gambiae not detected by PCR*^*2*^	1	10	9.1	0.1
*An. pharoensis*^*1*^	1	12	7.7	0.1
*An. coustanii*^*1*^	1	67	1.5	0.1
*An. quadriannulatus*^*2*^	1	85	1.2	0.1
*An. parensis*^*2*^	0	12	0.0	0.0
*An. vaneedeni*^*2*^	0	3	0.0	0.0
*An. gambiae* s.s.^*2*^	0	2	0.0	0.0
*An. maculpalpis*^*1*^	0	3	0.0	0.0
*An. nili*^*3*^	0	1	0.0	0.0
*An. pretoriensis*^*1*^	0	1	0.0	0.0
Total	130	947		12.1

^1^Identified morphologically

^2^Identified by the *An. gambiae* or the *An. funestus* complex PCR assay

^3^Identified by sequencing of the ribosomal DNA internal transcribed spacer region 2 (rDNA ITS2)

### Factors associated with bancroftian filariasis transmission

**Figure 4:**
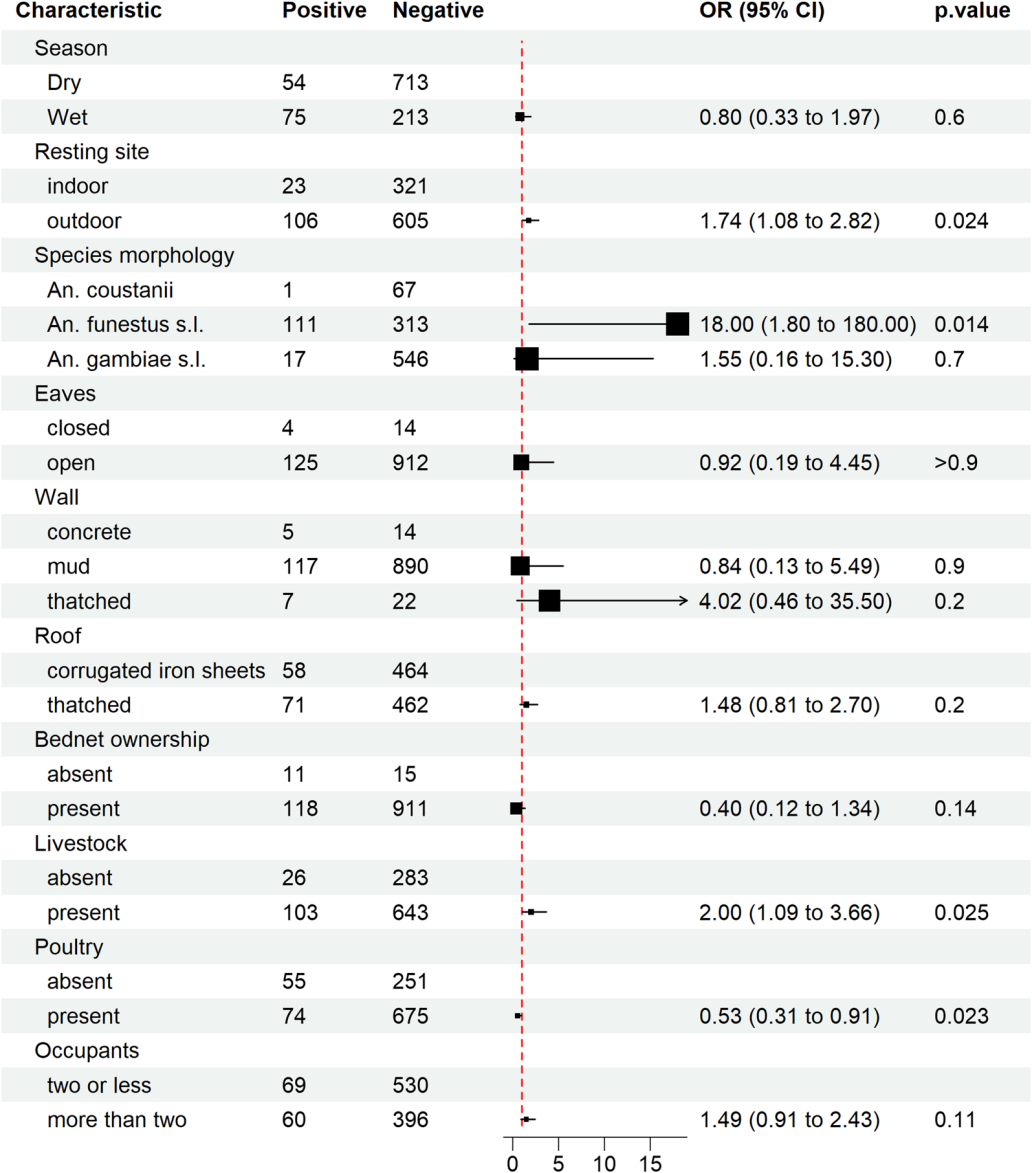
Multilevel logistic regression model based on generalized estimating equations on the various factors that may be associated with lymphatic filariasis transmission

The *An. funestus* s.l. mosquitoes exhibited a significant association with lymphatic filariasis transmission (OR 18.0; 95% CI 1.80–180, *p* = 0.014) (Fig. 4). Additionally, outdoor resting mosquitoes (OR 1.74; 95% CI 1.08–2.82, *p* = 0.024) and the presence of livestock around homesteads (OR 2.00; 95% CI 1.09–3.66, *p* = 0.025) were also significantly associated with LF transmission. Although households with thatched roofs showed increased odds of LF transmission, this association did not reach statistical significance. Conversely, poultry ownership demonstrated a significant reduction in the odds of LF transmission. While bed net ownership in the study area was associated with protection, this association did not attain statistical significance (OR 0.40; 95% CI 0.12–1.34, *p* = 0.14).

## Discussion

We investigated the vectorial systems for LF in rural coastal Kenya and factors associated with the risk of disease transmission in the region. Efforts to eliminate LF through MDA (albendazole and diethylcarbamazine citrate), as recommended by the WHO, began in the coastal region more than two decades ago. It was carried out in subsequent years and briefly interrupted by the coronavirus disease 2019 (COVID-19) pandemic. Various survey studies conducted during this period reported CFA prevalence rates ranging from 0.3% to 6.3% in Kwale, Kilifi, and Lamu counties; however, no LF cases were reported in Taita-Taveta county [7]. A mosquito survey in Malindi in 2012 showed very low prevalence, where only 1 out of 1055 pools of mosquitoes were positive for LF [33]. Using MX, we have demonstrated that there is active transmission of LF in Kilifi, Kwale, and Taita-Taveta counties warranting further MDA campaigns. The reasons for the persistent transmission are unclear but could be due to MDA adherence issues, therapeutic efficacy, or vector competence [7].

Malaria and LF are co-endemic in the Kenyan coast and are transmitted by similar vectors [11, 29]. Over the last two decades, campaigns to control malaria relying on long-lasting insecticide bed nets (LLINs) and indoor residual sprays (IRS) have been associated with decreased malaria incidence by limiting indoor biting and resting of anthropophilic vectors, thereby shifting vectors to more outdoor transmission [22]. This study reveals a higher prevalence of LF in outdoor resting mosquitoes, while LLIN ownership was associated with reduced risk of LF. This suggests that malaria interventions may alter LF transmission dynamics and complement MDA efforts [22, 34]. These findings call for the deployment of control interventions targeting outdoor resting mosquitoes such as attractive toxic sugar baits [35, 36], larvicides [37, 38], genetic vector control approaches [39], and endectocides [40, 41].

*An. funestus* s.l. and *An. gambiae* s.l. are both involved in the transmission of LF in the study area, with *An. funestus* s.l. playing a more significant role. In the *An. funestus* complex, *An. rivulorum* is the dominant vector of LF in counties adjacent to the Indian ocean, whereas *An. funestus* s.s. dominates further inland. In the *An. gambiae* complex, *An. arabiensis*, *An. merus*, and *An. quadriannulatus* were positive for LF without a clear regional preference. Other mosquito species, such as *An. coustanii* and *An. pharoensis*, were indicative of LF infection, although we had very few positive samples to draw substantive conclusions about their role in LF transmission. Despite observing high densities of *Cx. quinquefasciatus*, this study did not focus on this vector due to its well-understood role in LF transmission in urban areas [42, 43].

The presence of livestock in a homestead was strongly associated with LF transmission, suggesting that domestic animals play a critical role in sustaining LF vectors [34]. Similar observations have been made for *Aedes albopictus*, which disappeared with the elimination of rats, their preferred vertebrate host [44]. Therefore, incorporating vector control tools that restrict access to livestock can help suppress mosquito populations and potentially contribute to the elimination of lymphatic filariasis. Several studies are evaluating such tools, including endectocides such as ivermectin for controlling exophagic and zoophilic mosquitoes [45–47].

Houses with thatched roofs had increased odds of LF transmission, which is consistent with reports from India [48, 50]. These structures may provide favorable resting sites for mosquitoes. Therefore, improvements in house design to incorporate mosquito screens using relatively abundant and affordable materials, such as papyrus mats ceilings, have been shown to reduce *An. funestus* and *An. gambiae* entry by nearly 80% [49]. Similar modifications could be adopted to limit LF transmission in coastal Kenya.

Interestingly, poultry keeping was associated with a lower risk of LF infections, a phenomenon previously observed where *An. arabiensis*, despite opportunistically feeding on livestock, avoids chickens as potential source of bloodmeals. Factors contributing to this behavior include physical barrier such as chicken feathers, chicken predation on mosquitoes [50, 52], host-choice evolution driven by variation in the physical and chemical properties in the host blood, or a combination of these factors [51, 52]. Additionally, chickens produce volatiles such as isobutyl butanoate, naphthalene, hexadecane, and *trans*-limonene oxide, which repel mosquitoes [50]. Therefore, poultry keeping in peridomestic space may offer additional benefits by controlling mosquito vectors.

## Conclusions

In this study, we identified *An. funestus* s.l. sibling species, *An. rivulorum* and *An. funestus* s.s., as the dominant vectors of lymphatic filariasis along the Kenyan coast. We also showed that a higher proportion of transmission is likely to take place outdoors, necessitating the implementation of vector control strategies that target exophilic mosquitoes, such as zooprophylaxis and larval source management. We also showed the importance of MX in LF surveillance, as it is noninvasive and has the potential for incriminating new LF vectors.

## Data Availability

Most of the dataset used for analysis is available in the manuscript. We withheld the geo-data that may predispose individual homesteads to a high risk of identifiability. However, they are under the custodianship of the KEMRI-Wellcome Trust Data Governance Committee and are accessible upon request addressed to that committee.

## Abbreviations

LF: Lymphatic filariasis
MX: Molecular xenomonitoring
MDA: Mass drug administration
GPELF: Global programme to eliminate lymphatic filariasis
WHO: World Health Organization
*W.** bancrofti*: *Wuchereria bancrofti*
IRS: Indoor residual spray
LLINs: Long-lasting insecticide-treated nets
CFA: Circulating filarial antigen
geeglm: Generalized estimating equations generalized linear model
